# Striatal dopamine deficits predict reductions in striatal functional connectivity in major depression: a concurrent ^11^C-raclopride positron emission tomography and functional magnetic resonance imaging investigation

**DOI:** 10.1038/s41398-018-0316-2

**Published:** 2018-11-30

**Authors:** J. Paul Hamilton, Matthew D. Sacchet, Trine Hjørnevik, Frederick T. Chin, Bin Shen, Robin Kämpe, Jun Hyung Park, Brian D. Knutson, Leanne M. Williams, Nicholas Borg, Greg Zaharchuk, M. Catalina Camacho, Sean Mackey, Markus Heilig, Wayne C. Drevets, Gary H. Glover, Sanjiv S. Gambhir, Ian H. Gotlib

**Affiliations:** 1 Center for Social and Affective Neuroscience, Department of Clinical and Experimental Medicine, Linköping University, Linköping, Sweden; 2 Department of Psychiatry and Behavioral Sciences, Stanford University, Stanford, CA USA; 3 Department of Diagnostic Physics, Oslo University Hospital, Oslo, Norway; 4 The Norwegian Medical Cyclotron Centre, Oslo, Norway; 5 Department of Radiology, Stanford University, Stanford, CA USA; 6 Department of Psychology, Stanford University, Stanford, CA USA; 7 Center for Neuroscience, University of Pittsburgh, Pittsburgh, PA USA; 8 Department of Anesthesiology, Stanford University, Stanford, CA USA; 9 Janssen Research & Development, LLC, Johnson & Johnson, Titusville, NJ USA

## Abstract

Major depressive disorder (MDD) is characterized by the altered integration of reward histories and reduced responding of the striatum. We have posited that this reduced striatal activation in MDD is due to tonically decreased stimulation of striatal dopamine synapses which results in decremented propagation of information along the cortico-striatal-pallido-thalamic (CSPT) spiral. In the present investigation, we tested predictions of this formulation by conducting concurrent functional magnetic resonance imaging (fMRI) and ^11^C-raclopride positron emission tomography (PET) in depressed and control (CTL) participants. We scanned 16 depressed and 14 CTL participants with simultaneous fMRI and ^11^C-raclopride PET. We estimated raclopride binding potential (BP_ND_), voxel-wise, and compared MDD and CTL samples with respect to BP_ND_ in the striatum. Using striatal regions that showed significant between-group BP_ND_ differences as seeds, we conducted whole-brain functional connectivity analysis using the fMRI data and identified brain regions in each group in which connectivity with striatal seed regions scaled linearly with BP_ND_ from these regions. We observed increased BP_ND_ in the ventral striatum, bilaterally, and in the right dorsal striatum in the depressed participants. Further, we found that as BP_ND_ increased in both the left ventral striatum and right dorsal striatum in MDD, connectivity with the cortical targets of these regions (default-mode network and salience network, respectively) decreased. Deficits in stimulation of striatal dopamine receptors in MDD could account in part for the failure of transfer of information up the CSPT circuit in the pathophysiology of this disorder.

## Introduction

Researchers conducting neuroimaging-based investigations of major depressive disorder (MDD) have generated significant and useful brain-based conceptualizations of this illness. As the corpus of functional neuroimaging data from depressed samples has expanded, we have identified a variety of robust neural irregularities in MDD. A meta-analysis of results from functional connectivity studies of depression has, for example, identified reliable abnormalities in patterns of intrinsic functional connectivity in MDD^1^. In addition, multi-center integrations of functional neuroimaging data from depressed samples have allowed for neuro-typing that predicts response to treatment in MDD^2^. Further, researchers have developed neural-network-level conceptualizations of the effects of biologically-based therapies for depression^3,4^.

The new horizons afforded by developments in our brain-based understanding of depression have also generated novel questions about the neural bases of this disorder. One class of questions concerns the broader neurobiological contexts contributing to network-level disturbances in MDD. For example, in a meta-analysis of functional neuroimaging investigations of depression we identified a reliable failure of response of the striatum in MDD for both positively and negatively valenced stimuli^5^. Based on the valence-independent nature of this finding, in addition to observed decrements in the integration of reinforcement histories in MDD^6^, we proposed that there is a tonic deficit in dopaminergically-mediated neural transmission through the striatum in depression^5^. Data from radioligand binding imaging studies of striatal dopamine type-2 receptor function in MDD provide tentative backing of this formulation. Four studies have found reduced dopamine activity^7–10^, one found increased activity^11^, and seven found no differences in dopamine activity between depressed an healthy samples^12–17^. Interestingly, two of the four studies that found reduced dopamine activity in MDD incorporated a voxel-wise approach, similar to the one we use in the present investigation, while all others averaged across striatal regions of interest, potentially “averaging out” regional effects in the data.

We situate the current investigation in the context of the cortico-striatal-pallido-thalamic (CSPT) macro-architecture of the brain, a large-scale re-entrant circuit that was originally conceptualized as a motor circuit^18^. Subsequent work, however, showed that this circuit, and its cortical afferents and efferents, were roughly segregated in terms of limbic, cognitive, and motor functions^19^. In an important advance in our understanding of CSPT functioning, Haber and colleagues showed—based on extensive work with animal models—that the CSPT circuit is better conceptualized as an upward-projecting spiral in which information proceeds from limbic to cognitive to motor centers by way of dopaminergic projections to the striatum^20,21^. Therefore, we proposed that the failure of striatal and dorsal cortical response observed in depression could be due in part to a deficit in striatal dopaminergic stimulation in MDD that attenuates signal conduction up the CSPT spiral^5^.

In the present investigation, we used concurrent ^11^C-raclopride positron emission tomography (PET) and functional magnetic resonance imaging (fMRI) to test specific predictions extending from our prior formulation^5^. We predicted that: (1) we would replicate previous findings of decreased tonic striatal dopamine activity in MDD, indexed by the binding potential (BP_ND_) of ^11^C-raclopride; and (2) resting functional connectivity between striatal regions that showed reduced dopaminergic activity in depression and their cortical targets, measured with fMRI, would be reduced as a function of the decrement in striatal dopaminergic stimulation. For this study, we conceptualized cortical targets of striatal regions primarily in terms of large-scale intrinsic functional connectivity networks, such as default-mode, salience, and executive networks^22–24^. Additionally, we aimed to understand whether the reduction of striatal dopaminergic tone in MDD is due to reduced release of endogenous dopamine. Based on the success of previous work^25^, we addressed this aim by implementing a behavioral dopaminergic challenge: an extended block of the monetary incentive delay (MID) task^26^, shown reliably to elicit striatal response^27^. To accomplish both of these aims, we employed a novel design that incorporated a period of baseline scanning sufficient to determine basal BP_ND_ of raclopride followed by a period of behavioral challenge.

## Methods

### Participants

Sixteen individuals with current MDD (9 female, 7 male; average age = 32.77 years) and 14 healthy volunteers (CTL; 10 female, 4 male; average age = 32.46 years) were recruited. All participants were between the ages of 18 and 60 years, had no reported history of brain injury, had no reported substance abuse within the 6 months prior to assessment, and had no MRI contra-indications (e.g., implanted metal, claustrophobia).

All depressed participants met criteria for a DSM-5 diagnosis of MDD based on their responses to the Structured Clinical Interview for DSM^28^, administered by trained staff; none of the CTL participants met diagnostic criteria for any current or past psychiatric disorder. Depressed individuals who met criteria for a current diagnosis of any psychiatric disorder with the exception of an anxiety disorder were not included in the study. All participants completed the Beck Depression Inventory-II^29^ (BDI), the Hamilton Depression Rating Scale^30^ (HAM-D), the Beck Anxiety Inventory^31^ (BAI), and the Snaith–Hamilton Pleasure Scale^32^ (SHPS). Two depressed individuals were taking bupropion at the time of scanning; all other participants were, per self-report, not taking any psychoactive medications at the time of the scan. Further, two CTL and two MDD participants were taking birth-control drugs at the time of the scan. Written informed consent was obtained from participants prior to enrolling them in the study. The study protocol was approved by the Stanford University Institutional Review Board, and maintained compliance with federal, state, and local regulations on medical research (IND 123, 410).

### Concurrent PET-MRI scanning overview

**Fig. 1:**
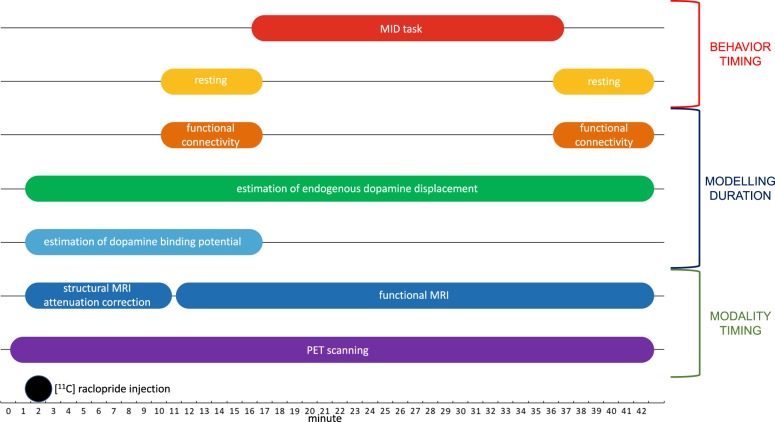
Schematic of the timing of data collection, data modelling, and participant behavior for our concurrent PET-MRI scanning paradigm

All participants underwent a simultaneous PET-MRI examination on a time-of-flight (TOF) PET-MRI scanner (SIGNA PET-MRI; GE Medical Systems, USA). PET scanning took place for the entirety of the 42-min scanning session. A bolus injection of ^11^C-raclopride was administered 1 min after the PET scan commenced and, at this same time, we began to obtain brain-structural and MRI-based attenuation correction data for 9 min. We then initiated a 32-min-long fMRI scan. Participants were instructed to rest with their eyes open for the first and last 6 minutes of this scan and to complete trials of a reward challenge task—the MID task, described below—for 20 min between these two rest conditions. Minutes 2–17 of PET data were used to estimate basal BP_ND_; PET data from Minute 2 onward (baseline plus task) were used to estimate endogenous ligand displacement by the task. See Fig. 1 for a depiction of the timing of data collection, data modelling, and participant behavior.

### PET data acquisition, pre-processing, and first-level analysis

#### Radiosynthesis and injection

^11^C-raclopride was prepared in a GE TRACERlab FX C Pro module (GE Healthcare). The approach we used was adapted from a method from Langer and colleagues^1^ with modifications specified in the following. Briefly, ^11^C-carbon dioxide was delivered from a GE PETtrace cyclotron (GE Healthcare) into the synthetic module, where the methylation agent ^11^C-methyl triflate was formed from ^11^C-carbon dioxide via reduction, halogenation, and triflation. ^11^C-methyl triflate was bubbled with a flow rate of 20 mL/min into solution containing acetone (300 µL), *O*-Desmethyl free base precursor (1 mg, 3.3 µmol, ABX GmbH), and NaOH (3 µL, 1 N) at −20 °C. The reaction mixture was warmed to room temperature within 1 min, diluted with 1 mL water, and loaded on a semi-prep HPLC for purification (Phenomenex Luna C18 5 µm, 250 × 10 mm, 30% acetonitrile, 70% 0.1 M NH_4_HCO_2_ with 0.5% AcOH, 7 mL/min). The fraction corresponding to ^11^C-raclopride (Rt = 9.2 min) was collected into a round-bottomed flask preloaded with 20 mL water. Mobile phase was then removed by solid phase extraction (SPE) and ^11^C-raclopride was eluted from the SPE cartridge with ethanol and subsequently diluted with saline (ethanol < 10% v/v). Final purified ^11^C-raclopride was sterilized by passing through a 0.22 µm Millex MP (33 mm) sterile filter. Overall synthesis time was 45 min and the radiochemical yield of ^11^C-raclopride was 1.4 ± 0.4% (*n* = 39). Analytical HPLC (Phenomenex Gemini C18 5 µm, 250 × 4.6 mm, 60% acetonitrile, 40% 0.1 M NH_4_HCO_2_ with 0.5% AcOH, 1 mL/min) showed the final product (Rt = 7.8 min) to have >99% radiochemical and chemical purity and molar activity 10.6 ± 3.9 Ci/µmol (392.2 ± 144.3 GBq/µmol; *n* = 39, decay corrected to end of synthesis). Synthesized ^11^C-raclopride was delivered to the scanning suite where participants were then injected via the left antecubital vein with 10 mCi over 60 s.

#### PET acquisition parameters

Listmode PET data were acquired for 42 min and dynamically reconstructed into 30-s time frames. The field-of-view (FOV) was 22 cm with a matrix size of 128 × 128. The PET data were reconstructed using a fully 3D TOF iterative ordered subsets expectation maximization algorithm (28 subsets, 3 iterations), and corrected for attenuation, scatter, point spread function, dead time, and decay. MR-based attenuation correction was performed with the clinical atlas-based method as implemented on the SIGNA PET/MR system, where individual T1-weighted MR images were rigidly and non-rigidly registered to a CT-based head atlas.

#### Estimating basal BP_ND_ of raclopride

For each participant, each 30-s frame of PET data was realigned to the summed image of the first 4 min of PET scanning. We then estimated BP_ND_ voxel-wise from PET time activity curves constructed from data acquired from Minute 2 to Minute 17 (see Fig. 1). Given that measuring BP_ND_ over shorter intervals renders small but reliable positive biases in BP_ND_ estimates^33^, we used the multilinear reference tissue model—implemented in PMOD 3.7 (http://www.pmod.com), with the cerebellum as the reference tissue—in the present analysis given that it has been shown to significantly reduce bias in BP_ND_ estimates^34^. Further, we conducted an independent validation test of our approach to estimating BP_ND_ by determining if, using this approach, we could replicate the reliable finding of reduced raclopride binding potential with advancing age^35–38^. To do this, we used BP_ND_ maps from all participants (i.e., collapsing across age-matched MDD and CTL groups) to obtain an age-by-BP_ND_ correlation map (family-wise error corrected at *α* = 0.05). Consistent with previous work, we observed a negative correlation between age and raclopride BP_ND_ in the putamen bilaterally; see Supplemental Figure 1.

#### Estimating task-induced ligand displacement

To quantify the magnitude of ligand displacement (and hence endogenous dopamine release) due to the performance of the MID task, we applied the linear simplified reference tissue model^39^ (LSRTM) to the motion-corrected dynamic data set. We used the LSRTM model to quantify the change of endogenous neurotransmitter level occurring immediately after task onset, in comparison to the 15-min baseline acquisition. Parametric images of the main outcome measure, the amplitude (*γ*) of ligand displacement, were computed using the start of task onset as an input parameter and the cerebellum as the reference tissue. Our implementation of the MID task for this study was designed to optimize sensitivity for detection of ligand displacement, not BOLD signal change. Each participant completed 160, 7.5 s trials (3 TRs, 2.5 s each) of the MID task. Eighty-two trials (51.2%) involved non-gain stakes of ±$0; 39 trials (24.4%) incorporated low-grain stakes of±$1; and 39 trials (24.4%) involved high stakes of ±$5. All participants began the task with $10 in winnings. Each trial began with a 2-s cue period during which participants saw an unfilled circle (indicating a gain trial) or an unfilled square (indicating a loss trial) with horizontal lines within the shape indicating reward magnitude (0 lines for non-gain/loss trials and one or three lines for $1 or $5 gain/losses, respectively). This was followed by a 1.5–2.5 s target anticipation period, during which participants saw a plus sign. In the following target response period, participants saw a filled white square and pressed a button to attempt to win or avoid losing money—the duration of the filled white square was dynamically adjusted to keep the rate of success at about 67%. This was followed by an outcome-information period of approximately 2 s during which participants saw their current winnings or losses and new total balance.

### MRI data acquisition, preprocessing, and first-level analysis

#### MRI acquisition parameters

Whole-brain fMRI data were collected with the following specifications: FOV = 220 mm, matrix = 64 × 64, through-plane resolution = 3.5 mm, in-plane resolution = 3.44 × 3.44 mm, slice spacing = 0, TR = 2500 ms, TE = 30 ms, flip angle = 80°, slices = 27 sequential ascending/axial. High-resolution spoiled-gradient echo T1-weighted anatomical images were collected using the following parameters: FOV = 220 mm, matrix = 256 × 256, through-plane resolution = 1.2 mm, in-plane resolution = 0.86 × 0.86 mm, slice spacing = 0, TR = 8.52 ms, TE = 3.32 ms, TI = 450 ms, flip angle = 12°, slices = 124 acquired sequentially left-to-right in sagittal plane.

#### Preprocessing of resting fMRI data

Working within the Analysis of Functional NeuroImages^2^ (AFNI) platform, we applied to each 6-min epoch of eyes-open, resting fMRI data despiking followed by slice-time correction and then spatial co-registration to the first image from each respective epoch. We then applied a procedure for regression-based, low-pass filtering and an algorithm for correction of measurement artefacts^3^. Further, translational and rotational motion regressors, their first derivatives, and polynomial drift regressors in addition to regressors for unmodeled residual noise^4^ were applied to the data as noise covariates.

#### Seed-based functional connectivity analysis

Using the cleaned resting fMRI data, we conducted seed-based functional connectivity analysis for each participant. We entered time-series data from three striatal regions in which we observed significant differences in basal BP_ND_ between MDD and CTL samples (see below) into separate regressions against whole-brain data. Within these regression analyses, we applied a censoring/scrubbing procedure for which functional acquisitions (i.e., TRs) in which participant motion exceeded 0.2 mm were censored from the regression. A given 6-min resting-state fMRI epoch was removed from further consideration if participant motion exceeded 0.2 mm for 25% or more of the functional acquisitions.

### Integrating cross-modal data and second-level analyses

#### Spatial alignment

It was important for the present multi-modal imaging study that data from PET and fMRI modalities were aligned and co-registered as precisely as possible in a common stereotaxic space. To achieve this, we sought first to ensure that both fMRI and PET data were well aligned with high-resolution structural data in native space. To the first acquisition of fMRI data for each 6-min scan and to the high-resolution structural data, we applied AFNI’s 3dUnifize for maximizing contrast between grey and white matter regions for achieving better cross-modal alignment. Next, we applied the AFNI procedure align_epi_anat.py to align the high-contrast fMRI data with the high-contrast structural data. For the PET data, a visual check of the first acquired frames relative to the brain structural data indicated excellent native space alignment without further processing. Given that the binding potential of ^11^C-raclopride is selectively high throughout the striatum, we confirmed this again by examining individual BP_ND_ maps relative to the striatum as rendered in the high-resolution structural data. Finally, we determined optimum affine and non-linear warping parameters to standard space^40^ using the high-resolution structural data and then applied these same parameters to the aligned fMRI and PET data.

#### Voxel-wise group comparisons BP_ND_ and γ

To compare groups with respect to BP_ND_ we first defined a striatal region of interest for which average BP_ND_ ≥ 1 across participants’ spatially co-registered data and compared groups (family-wise error corrected at *α* = 0.05 as determined from AFNI’s 3dclustsim) voxel-wise within this mask using AFNI’s 3dttest++. We applied this same procedure to comparing groups voxel-wise with respect to γ, the index of task-related ligand displacement. Normality tests of whole-brain, voxel-wise data were conducted using AFNI’s 3dNormalityTest. Given that sample sizes tend to be smaller for studies incorporating invasive procedures like PET, we did not include demographic variables as covariates in our group comparisons in order to preserve degrees of freedom. Instead, we matched groups as closely as possible with respect to demographic variables, particularly variables such as age^38^ and smoking^41^ that have been shown to affect dopamine activity.

#### Binding-potential-by-functional-connectivity correlations

Using as seeds peak regions in the three parts of the striatum—resampled to fMRI spatial resolution—in which significant between-group differences in BP_ND_ were detected, we performed whole-brain functional connectivity analysis (see above). Using AFNI’s 3dttest++, we identified in each group separately regions for which functional connectivity with a given seed region correlated significantly (family-wise error corrected at *α* = 0.05, as determined from AFNI’s 3dclustsim) with BP_ND_ estimates from that same region. We transformed these correlation coefficients into *z*-scores to allow for direct comparison of correlations across groups. Normality tests of whole-brain, voxel-wise data were conducted using AFNI’s 3dNormalityTest. As the spatial smoothness of neuroimaging data impacts the calculation of cluster thresholds, we mention here work indicating that, for block designs (and less so for event-related designs), AFNI’s traditional cluster thresholding approach can identify elevated levels of false-positive results^42^. While this formulation has not been tested in the context of functional connectivity analyses using resting fMRI data, we nonetheless indicate in Results whether correlation clusters are significant at more stringent (ACF) and/or conventional (FWHM) cluster thresholds.

#### Functional cortical projections of striatal seed regions

For descriptive purposes, we sought to identify the intrinsic functional network(s) to which the striatal seed regions defined by our between-group, voxel-wise comparison of BP_ND_ were functionally connected. To do this, we used data from the 1000 Functional Connectomes Project (http://fcon_1000.projects.nitrc.org) as processed and rendered in Neurosynth (www.neurosynth.org). Within Neurosynth’s functional connectivity interface, we entered as seed regions each of the three peak regions from the between-groups BP_ND_ comparison. We then warped the three resulting correlation maps from MNI to Talairach space, thresholded each map at *α* = 0.05, two-tailed, and then visually compared each statistical map to maps generated by Yeo and colleagues^5^; liberal, seven-network solution.

#### Code availability

All computer code used to generate the results reported here is freely available and can be accessed by contacting the corresponding author of this article.

## Results

### Sample characteristics

Demographic and clinical characteristics of the MDD and CTL samples, excluding one MDD participant whose data were removed from further consideration due to excessive motion during both 6-min resting fMRI epochs, are presented in Table 1. There were no significant group differences in gender composition, handedness, age, years of formal education, or motion during scanning. The MDD participants reported significantly higher levels of depressive symptomatology, as indexed by both the BDI and HAM-D, anxiety, as assessed with the BAI, and anhedonia, as indexed by the SHPS.

**Table 1:** Demographic, clinical, and experimental characteristics of samples

	MDD	CTL	*t* or *X*^*2*^	*p*
Gender composition (proportion female)	0.53	0.71	1.00	>0.10
Proportion right-handed	0.93	0.71	2.44	>0.10
Age (in years)	32.46 ± 2.37	33.22 ± 3.14	0.19	>0.10
Years of formal education	16.27 ± 0.55	16.29 ± 0.72	0.02	>0.10
Percentage fMRI acquisitions >0.2 mm motion	7.9 ± 0.02	11.1 ± 0.03	0.93	>0.10
Smoking (cigarettes per week)	0.3 ± 0.3	0.07 ± 0.07	0.72	>0.10
BDI	26.27 ± 2.69	1.07 ± 0.38	8.96	<0.05
HAM-D	13.6 ± 1.56	1.14 ± 0.46	7.46	<0.05
BAI	13.27 ± 2.15	1.71 ± 0.67	4.97	<0.05
SHPS	49 ± 2.34	64 ± 1.56	5.32	<0.05
Length of current depressive episode (months)	26.06 ± 13.10			
Lifetime number of depressive episodes	13.66 ± 3.21			
Time since onset of first depressive episode (years)	14.19 ± 2.64			
Proportion receiving psychotropic medication	0.13			
Proportion with a current comorbid anxiety disorder	0.20			

Error estimates are in standard error about the mean

*MDD* major depressive disorder, *CTL* healthy controls, *fMRI* functional magnetic resonance imaging, *BDI* Beck Depression Inventory, *HAM-D* Hamilton Depression Rating Scale, *BAI* Beck Anxiety Inventory, *SHPS* Snaith–Hamilton Pleasure Scale

Two depressed participants were taking psychotropic medication (bupropion) at the time of the scan. Given the action of bupropion as a norepinephrine-dopamine reuptake inhibitor, we examined our neuroimaging results without including participants who were taking bupropion^1^. We also analyzed our neuroimaging data excluding two light smokers (one each in CTL and MDD groups). All effects remained significant when medicated MDD and smokers were excluded. See Supplemental Figure 2.

### Administered mass and activity

The mean and standard error of the administered mass and activity of ^11^C-raclopride were 2.45 ± 0.39 µg (range: 0.80–6.09 µg) and 556.37 ± 9.74 MBq (range: 340.77–616.05 MBq) for the MDD patients, and 2.40 ± 0.19 µg (range: 1.16–3.63 µg) and 535.17 ± 16.92 MBq (range: 475.45–610.50 MBq) for healthy controls. The groups did not differ significantly with respect to administered mass or activity; *t* = 0.12 and 1.07, respectively, both *p* > 0.10.

### Baseline ^11^C-raclopride BP

**Fig. 2:**
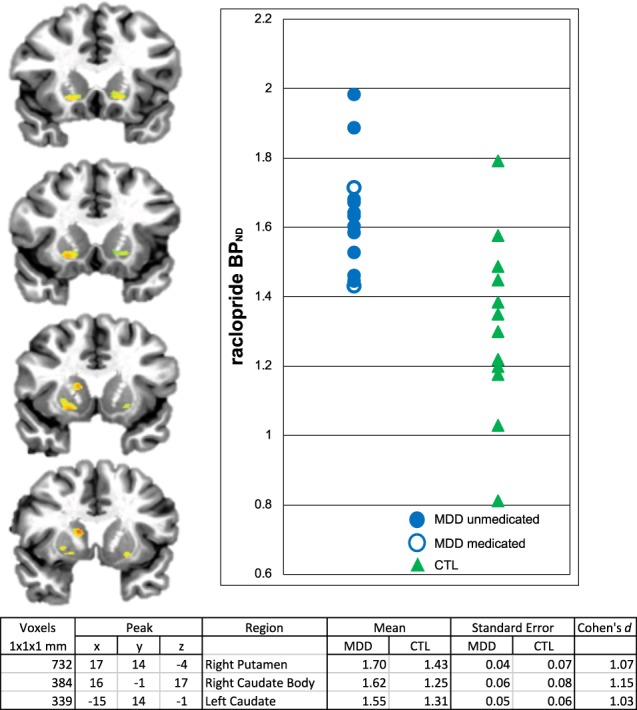
Statistical map, and corresponding cluster table, showing regions of increased ^11^C-raclopride binding potential in the depressed relative to the control group

We found higher basal BP_ND_ in depressed than in healthy participants in the ventral striatum, bilaterally, and in the right dorsal striatum. See Fig. 2 for a statistical map and cluster characteristics.

### Task-based modulation of ^11^C-raclopride time–activity curves

While we did observe across all participants reliable effects of MID-task performance relative to baseline on ^11^C-raclopride time–activity curves (see Supplemental Figure 3), the depressed and nondepressed groups did not differ significantly with respect to these effects.

### Correlations between baseline BP_ND_ and seed-based functional connectivity

#### Left ventral striatum

**Fig. 3:**
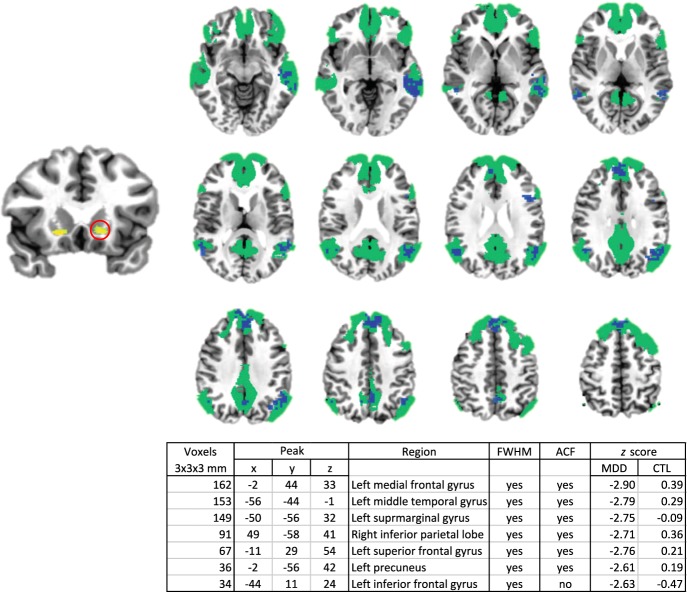
Left ventral striatal binding potential-by-functional connectivity correlation map. Regions showing statistically significant negative binding potential-by-functional connectivity correlations in the depressed group are shown in blue. These regions are superimposed on a map of the default-mode network (in green) as defined by Yeo and colleagues^5^. For convenience, the left ventral striatal seed region is shown at left.

For the left ventral striatal region showing increased BP_ND_ in MDD, we found in the MDD group that as BP_ND_ increased, functional connectivity between this region and several nodes of the default-mode network decreased. We found no significant BP_ND_-by-functional connectivity correlations in the CTL group. See Fig. 3 for a statistical map, cluster characteristics, and between-group cluster-wise comparisons showing significant correlation differences between groups. See Supplemental Figure 4.A. for a map verifying that the primary cortical projection of the left ventral striatal seed region is the default-mode network.

#### Right ventral striatum

For the right ventral striatal region that showed increased BP_ND_ in MDD, we found no significant BP_ND_-by-functional connectivity correlations in either group.

#### Right dorsal striatum

**Fig. 4:**
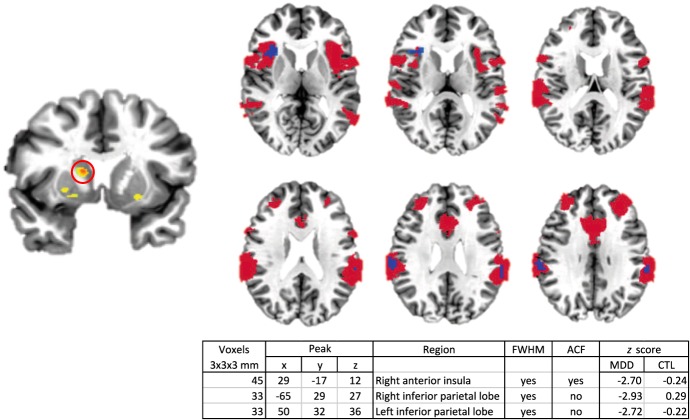
Right dorsal striatal binding potential-by-functional connectivity correlation map. Regions showing statistically significant negative binding potential-by-functional connectivity correlations in the depressed group are shown in blue. These regions are superimposed on a map of the task-positive network (salience and executive networks, in red) as defined by Yeo and colleagues^5^. For convenience, the right dorsal striatal seed region is shown at left

For the right dorsal striatal region in which we observed increased BP_ND_ in MDD, we found in the MDD group a negative relation between BP_ND_ in this region and functional connectivity with three nodes of the salience network. We found no significant BP_ND_-by-functional connectivity correlations in the CTL group. See Fig. 4 for a statistical map, cluster characteristics, and between-group cluster-wise comparisons showing significant between-group correlation differences. We show in Supplemental Figure 4.B. a map verifying that the primary cortical projections of the right dorsal striatal seed region are the salience network and executive network—together commonly referred to as the task-positive network.

## Discussion

In this investigation, we used concurrent ^11^C-raclopride PET and fMRI to test the hypothesis that reduced tonic striatal dopamine activity in depression—indexed by the increased binding potential of ^11^C-raclopride—would predict reduced functional connectivity between striatal regions showing a dopaminergic decrement in MDD and their cortical target regions. We found increased ^11^C-raclopride binding potential in both the left and right ventral striatum and right dorsal striatum in MDD. Moreover, and also consistent with our hypothesis, we found in the left ventral striatum and right dorsal striatum that increasing BP_ND_ of ^11^C-raclopride predicted decreasing functional connectivity between these regions and their respective default-mode and salience network targets in MDD.

Empirically-based cortico-limbic dysconnectivity hypotheses of depression^43^ that propose increased contributions from limbic regions and decreased contributions from dorsal cortical regions in MDD represent crucial advances to our neural-level understanding of depression but have lacked an explicit and mechanistic neurobiological hypothesis of this disconnection. Within the framework of the ascending spiral CSPT architecture of neural information flow^21^, our present findings indicate that reduced striatal dopaminergic activity and associated reductions in connectivity between striatal regions and their cortical targets may provide a partial, neurobiologically plausible account of this disconnection. Future work incorporating both striatal dopamine imaging and imaging of basal brain activity—indexed, for example, by resting regional glucose metabolism, which has contributed most directly to cortico-limbic models of MDD—could provide convergent validation of this partial account.

Beyond their implications for cortico-limbic models of MDD, the present multi-modal neuroimaging data suggest more generally that we can begin to understand intrinsic functional connectivity in the affective-motivational context of the ascending CSPT spiral model^5,20^. Investigations examining functional striatal–cortical connectivity have made critical contributions to our understanding of the CSPT architecture in humans^44^ but have not been conducted in the framework of intrinsic functional connectivity networks, which are now well-defined^24,45,46^. In showing both that patterns of striatal functional connectivity map well onto intrinsic functional connectivity networks and that this connectivity scales with striatal dopaminergic tone in MDD, the present investigation suggests that it is feasible to integrate what we know about the ascending spiral CSPT architecture and intrinsic functional connectivity networks in generating more comprehensive neural models of goal-driven behavior.

One limitation of the current study is the fundamental ambiguity of studying neurotransmitter dynamics via radiotracer imaging. In the context of our study, the finding of increased basal raclopride binding potential in MDD could be due either to reduced competition with endogenous ligand or to increased affinity of D_2_ receptors in MDD. To help distinguish between these possibilities, we introduced a behavioral dopaminergic challenge into our scanning paradigm but the result was inconclusive, possibly because our behavioral challenge was not sufficiently strong relative to pharmacological challenges used in previous investigations that have demonstrated raclopride-binding dynamics^47^. Another limitation of the current study is the relatively short baseline period we used in estimating binding potential. Previous work with ^11^C-raclopride has shown that shorter periods for modeling binding potential result in modest but reliable positive biases in binding-potential estimates^33^—an effect we attempted to ameliorate in the current investigation by applying the multilinear reference tissue model in estimating binding potential. Further, in the current study we did not obtain lifetime psychotropic medication histories for depressed participants. In juvenile monkeys, fluoxetine (a selective serotonin reuptake inhibitor) has been shown to have effects on serotonergic markers well after discontinuation^48^. This finding, considered in light of evidence of interactions between dopaminergic and serotonergic systems^49^ leaves open the possibility that the dopaminergic effects we report reflect long-term serotonergic effects. Finally, it is important to note that in terms of cortico-limbic dysconnectivity models of MDD, the present dopaminergic model is likely only one of several molecular-level moderators of functional connectivity in MDD.

In this study, we demonstrated that increased binding potential of raclopride in the striatum in depression predicts reductions in functional connectivity between affected striatal areas and their respective cortical targets. Dopaminergic therapies for depression have not been strongly pursued, likely because dopaminergic decrements in MDD are focal whereas dopaminergic interventions act more broadly and have negative side-effects^50^. Understanding the neural-functional implications of focal disturbances in dopaminergic tone, however, suggests novel routes through which the potential consequences of altered dopaminergic function could be addressed. For example, given the current results, investigators may seek to develop neurofeedback therapies that target connectivity between the ventral striatum and default-mode network as a neural-functional proxy of disordered dopaminergic tone in MDD.

## Electronic supplementary material

Supplemental Information

## Footnotes

1 Given our hypothesis that there would be increased binding of striatal ^11^C-raclopride in MDD, including depressed participants who are pharmacologically advantaged toward increased levels of post-synaptic dopamine makes our test of this hypothesis more conservative.
