# Supplementary material for: Striatal dopamine deficits predict reductions in striatal functional connectivity in major depression: a concurrent 11C-raclopride positron emission tomography and functional magnetic resonance imaging investigation
Source: Transl Psychiatry. 2018 Nov 30;8:264. doi: 10.1038/s41398-018-0316-2 (PMC6269434; doi:10.1038/s41398-018-0316-2)
Supplement: Supplementary file 1 — Supplemental Information [file 41398_2018_316_MOESM1_ESM.docx]

Supplemental Information

Results


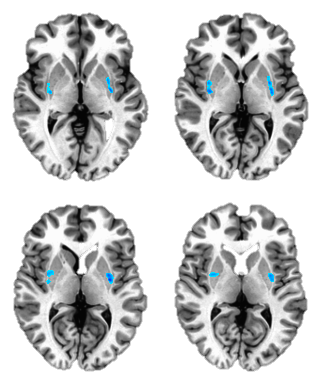


*Supplemental Figure 1.* Correlation map (*p* < 0.05, FWE corrected) depicting negative relation between age and baseline ^11^C-raclopride BP_ND_ in the putamen bilaterally across the full study sample.

*Supplemental Figure 2.* Statistical maps resulting from the study’s primary analyses (presented along the horizontal axis) excluding two patients taking bupropion (bottom row) and two light smokers (middle row) in contrast to results from the full study sample (top row).

*Supplemental Figure 3.* One-sample *t*-test map (*p* < 0.05, FWE corrected) comparing all participants’ γ coefficient maps to zero. The γ coefficient is an index of the amplitude of task-induced ligand displacement.

*Supplemental Figure 4.A.* Correlation map of left ventral striatal seed region in orange; default-mode network as determined by Yeo and colleagues (2) in yellow; and their intersection in red. *B*. Correlation map of right dorsal striatal seed region in orange; task positive network (salience and executive networks) as determined by Yeo and colleagues (2) in yellow; and their intersection in red.

**Cross epoch functional connectivity changes.** Given that connectivity can change across data-collection epochs and even within a given scanning run^6^, we compared connectivity estimates between the first and second epochs of resting fMRI scanning using both seeds for which we found BP_ND_ by connectivity correlation differences in MDD and CTL groups. Using a liberal statistical threshold (voxel-wise *p* = 0.05; *k* = 52 voxels), we found no cross-epoch differences for the left ventral striatal seed region. We did, however, identify two regions in which connectivity to the right dorsal striatal seed was greater in the first and second epoch: 1) left transverse temporal gyrus (center of mass = -50, -24, 13); 2); and 2) left cuneus (center of mass = -8, -78, 27). These regions did not intersect with regions showing BP_ND_ by right dorsal striatum connectivity correlation differences in MDD and CTL groups.

References

1. Langer O, Nagren K, Dolle F, Lundkvist C, Sandell J, Swahn CG *et al.* Precursor synthesis and radiolabelling of the dopamine D-2 receptor ligand C-11 raclopride from C-11 methyl triflate. *Journal of Labelled Compounds & Radiopharmaceuticals* 1999; **42**(12)**:** 1183-1193.

2. Cox RW. AFNI: software for analysis and visualization of functional magnetic resonance neuroimages. *Computers and Biomedical research* 1996; **29**(3)**:** 162-173.

3. Jo HJ, Saad ZS, Simmons WK, Milbury LA, Cox RW. Mapping sources of correlation in resting state FMRI, with artifact detection and removal. *Neuroimage* 2010; **52**(2)**:** 571-582.

4. Marx M, Pauly KB, Chang C. A novel approach for global noise reduction in resting-state fMRI: APPLECOR. *Neuroimage* 2013; **64:** 19-31.

5. Yeo BT, Krienen FM, Sepulcre J, Sabuncu MR, Lashkari D, Hollinshead M *et al.* The organization of the human cerebral cortex estimated by intrinsic functional connectivity. *Journal of neurophysiology* 2011; **106**(3)**:** 1125-1165.

6. Chang C, Glover GH. Time-frequency dynamics of resting-state brain connectivity measured with fMRI. *Neuroimage* 2010; **50**(1)**:** 81-98.
